# Influence of endurance versus resistance exercise training on central and peripheral chemoreflexes in young healthy individuals

**DOI:** 10.1016/j.jphyss.2025.100027

**Published:** 2025-05-15

**Authors:** Thalia Babbage, Ana L.C. Sayegh, Jui-Lin Fan, Nicholas Gant, Julian F.R. Paton, James P. Fisher

**Affiliations:** a Department of Physiology, Manaaki Manawa – The Centre for Heart Research, Faculty of Medical & Health Sciences, University of Auckland, New Zealand; b Department of Anaesthesiology, Faculty of Medical & Health Sciences, University of Auckland, New Zealand; c Department of Exercise Sciences, Faculty of Science, University of Auckland, New Zealand

**Keywords:** Exercise, Chemoreflex, Cerebral blood flow, Ventilation, Human

## Abstract

Heightened central and peripheral chemoreflex sensitivity are associated with poor outcomes, but therapeutic approaches to target them are lacking. Endurance and resistance exercise training improve a multitude of physiological outcomes, but their effects on ventilatory chemoreflex sensitivity are unclear. Accordingly, the cardiorespiratory responses to steady-state isocapnic hypoxia (10 % O_2_, 5-minutes) and hyperoxic hypercapnic rebreathing (5 % CO_2_-95 % O_2_) were compared in endurance, resistance, and untrained groups. Central chemoreflex sensitivity was taken as the slope of the relationship between minute ventilation (V̇_E_) and end-tidal partial pressure of CO_2_. Peripheral chemoreflex sensitivity was determined from the absolute increase in V̇_E_ from baseline to peak V̇_E_ expressed relative to the fall in oxygen saturation. Neither central (P = 0.093) nor peripheral (P = 0.847) ventilatory chemoreflex sensitivities were different between groups. Future investigations should seek to understand whether exercise training modality influences central and peripheral chemoreflex sensitivity in older and clinical populations.

## Highlights

• Heightened chemoreflex sensitivity is associated with poor outcomes.
• Exercise training improves central and peripheral chemoreflex sensitivity in animals.
• Central chemoreflex (hypercapnia) responses were not different in young humans.
• Peripheral chemoreflex (hypoxia) responses were also not different in young humans.

## Background

Enhanced central and peripheral chemoreflex sensitivities are common in cardiovascular conditions (e.g., heart failure) [1] and are associated with disease progression and mortality 2, 3. However, therapeutic options to specifically target chemoreflex sensitivity are currently limited. Animal models have identified that exercise training reduces resting [4] and peripheral chemoreflex-mediated [5] elevations in renal sympathetic nerve activity and blood pressure [6]. The underlying mechanisms include favourable changes in brainstem neural plasticity that lower sympathetic outflow 7, 8, and increased carotid body blood flow [9]. In contrast, human studies are equivocal regarding the effect of exercise training on peripheral and central chemoreflex sensitivity 10, 11, 12, 13, 14.

Aerobic and resistance exercise provoke distinct acute cardiorespiratory adjustments, along with longer-term cardiovascular and performance adaptations to training (increased endurance capacity versus increased muscle mass/strength) 15, 16, 17, 18, 19, 20. The ventilatory response to aerobic exercise is comprised of a fast initial response, slow exponential rise, followed by attainment of a steady level [21]. In contrast, the acute respiratory response to resistance training is cyclical with phase of contraction and no steady-state is reached [22]. Notably, metrics of cardiovascular autonomic function (e.g., heart rate variability) are improved with aerobic exercise training [23], while resistance exercise appears to have minimal effect on cardiovascular autonomic control 24, 25. The effect of endurance or mixed endurance and resistance exercise training interventions on chemoreflex function (either peripheral or central) have been assessed in both humans 10, 11, 12, 13, 14 and animal models 5, 6, 26, 27. However, the findings are mixed, with reports of both reductions 5, 6, 11, 13, 14, 26, 27 and no effect 10, 12, 13 on central and peripheral chemoreflex function. Therefore, it is currently unclear whether exercise training modality (i.e., endurance vs. resistance) influences peripheral and central chemoreflex sensitivity.

The experimental methods used to assess central and peripheral chemoreflexes in humans (e.g., administration of hypercapnia and hypoxia, respectively 28, 29) can also be used to determine cerebrovascular function where a concomitant assessment of cerebral perfusion is included [30]. This is relevant in the context of the current study because habitual exercise training and cardiorespiratory fitness have been well-documented for their neuro- and cardioprotective effects in relation to the development of conditions such as dementia and stroke 31, 32, 33. The underlying mechanisms are multifaceted and include positive effects on neurotrophic, metabolic and inflammatory factors along with enhanced cerebrovascular structure and function 34, 35. Key cerebrovascular mechanisms include the upregulation of endothelial derived nitric oxide signalling (i.e., enhanced endothelial function) associated with exercise-induced increases in shear stress, and lowered arterial stiffness (i.e., enhanced vascular compliance) that can reduce pulsatility within the cerebral microvasculature 34, 35. Moreover, exercise-induced increases in carotid body blood flow, secondary to increased cerebral perfusion and/or lowered sympathetic activity, have the potential to lower peripheral chemoreflex sensitivity [36]. Of note, the cerebrovascular stimulus is dependent on exercise modality; a constant shear stress on the cerebral vasculature during steady state aerobic training versus intermittent surges in shear stress during periods of loading with resistance training [37]. Metrics of cerebrovascular function (cerebrovascular reactivity [CVR_CO2_] and pulsatility index) have been assessed in response to exercise training interventions [38], and cross sectionally 39, 40. However, previous studies have not assessed cerebrovascular responses to both hypoxia and hypercapnia in habitually resistance or endurance trained athletes. Therefore, it remains unclear whether habitual resistance versus endurance exercise training differentially affects CVR_CO2_ and pulsatility index in young, healthy adults and if this correlates with changes in central and /or peripheral chemoreflex sensitivity.

The primary aim of the present study was to determine the influence of endurance and resistance exercise training on the central and peripheral chemoreflexes, using hyperoxic hypercapnia and isocapnic hypoxia, respectively. It was hypothesised that exercise trained individuals would have lower central and peripheral chemoreflex sensitivities than their untrained counterparts, and that chemoreflex sensitivity will be lower in endurance versus resistance trained individuals. The secondary aim was to assess the cerebrovascular responses to hyperoxic hypercapnia and isocapnic hypoxia in endurance, resistance and untrained groups. It was hypothesised that trained individuals, particularly endurance trained individuals, will have superior cerebrovascular reactivity/pulsatility. A cross-sectional study design was utilised as it was believed to be an important first step, and one which permits the recruitment of participants who had been doing specific training for at least 12 months.

## Methods

### Study participants

Ten untrained (5 men), twelve endurance-trained (7 men) and twelve resistance-trained (7 men) young healthy adults were recruited for this study. All participants were free of any chronic medical conditions (cardiovascular, respiratory, metabolic or neurological). Participants arrived at the laboratory having abstained from food for 2 h prior to the study, caffeine and alcohol for 12 h prior to the study, exercise after 8:00 pm the evening prior to the study or on the day of the study, and ‘over the counter’ medications on the day of the study. Participants self-reported their exercise training status at the Familiarisation visit. Self-reported exercise training history was classified as: participating in 4 or more hours per week of either aerobic training or resistance training, on two or more days per week, for a continuous period of > 12 months, or not engaging in any regular physical activity. Individuals with a BMI < 18 kg.m^−2^, those who were current smokers, users of recreational drugs or abusers of alcohol, or who had any underlying medical conditions or current pregnancy were excluded. Women performed the experimental visit (chemoreflex assessment) during the first five days of their menstrual cycle (early follicular phase, n = 10), during the placebo/no-hormone phase of combined oral contraceptive use (n = 1) or scheduled at any time if taking a progesterone only oral contraceptive or Mirena intra-uterine device (n = 4).

### Experimental design

Participants attended the laboratory on two separate occasions separated by at least 48 h, for a familiarisation visit and an experimental visit. The familiarisation visit involved categorisation of participants by training status with a measure of handgrip strength and a graded treadmill maximal exercise test, as well as familiarisation of participants to study equipment and protocols.

### Familiarisation visit protocol

Handgrip strength: Participants were familiarised with the handgrip dynamometer (TTM Digital Hand Dynamometer, Japfollan) and used their dominant hand to perform a maximal voluntary contraction (MVC). MVC was repeated 3 times to attain values within 5 % of each other, with further trials performed util 3 reproducible values were obtained (up to a maximum of 6 trials). The highest value was reported.

Graded maximal exercise test: V̇O_2_peak was determined using a graded treadmill (TechnoGym Excite 500i, TechnoGym, Italy) maximal exercise test. Participants were instrumented with a Polar chest strap heart rate monitor (Polar H10, Polar Electro, Finland), head piece, T-shape two-way non-rebreathing valve (Hans Rudolph Inc., Shawnee, KS, USA) and nose-clip for collection of expired gas. The outlet of the two-way valve was attached via wide bore tube to a mixing chamber. A pneumotachometer (Respiratory flow Head 1000 L, MLT1000L, ADInstruments, New Zealand) attached to the opposite end of the mixing chamber measured expiratory flow and sampling line attached near the pneumotach extracted a gas sample for analysis (Respiratory Gas Analyser, ML206, ADInstruments). A thermistor probe was also connected at the point of gas sampling for expired air temperature correction. Treadmill speed remained constant with gradient increased by 2 % every 2-minutes similar to that described by Lundby et al. [41] until volitional exhaustion. Immediately following test termination, a rating of perceived exertion (RPE) score on a 0–10 Borg scale [42] was obtained. Achievement of a maximal exercise test was quantified using the following criteria: respiratory exchange ratio (RER) ≥ 1.10, rating of perceived exertion (RPE) ≥ 9, and age-predicted heart rate maximum (APHRM) ≥ 85 % 43, 44.

### Experimental visit protocol

Participants attended the laboratory during normal work hours (0900–1700). At the experimental visit, participants performed two gas challenges (hyperoxic hypercapnia followed by isocapnic hypoxia). Importantly, the order of gas challenges was not randomised due to the potential for complete respiratory recovery from hypoxia to take up to 60 min 45, 46. Hyperoxic hypercapnic rebreathing was performed according to Duffin’s modified rebreathing method [28]. Briefly, participants maintained restful breathing for a baseline of 5 min, followed by hyperventilation to reduce their end tidal partial pressure of CO_2_ (P_ET_CO_2_) to ∼25 mmHg, before exhaling to below functional residual capacity. Participants were then switched to a closed-circuit breathing system containing 95 % O_2_-5 % CO_2_ and took three rapid, deep breaths. Participants then rebreathed from the circuit until P_ET_CO_2_ ∼55 mmHg was achieved. A washout period of 10-mins was observed, followed by a second 5-min baseline of restful breathing. Participants were then switched to a two-way non-rebreathing valve with inspiratory flow from a cylinder and 3 L reservoir containing 10 % O_2_ (balance N_2_) to maintain end tidal partial pressure of O_2_ (P_ET_O_2_) ∼50 mmHg. Isocapnic hypoxia was maintained for 5 min

### Experimental measures

Heart rate (HR) was monitored with a lead II electrocardiogram (ECG, BioAmp, FE231, ADInstruments, Bella Vista, NSW, Australia) and beat-to-beat blood pressure (BP) measured using finger photoplethysmography (Finapres Nova, Finapres Medical Systems, Enschede, Netherlands). Finger BP values were calibrated internally within the Finapres Nova device using brachial artery BP measurements. Arterial oxygen saturation (S_P_O_2_) was measured using finger pulse oximetry (MLT320/F and MLT321; ADInstruments). Transcranial Doppler ultrasonography of the right middle cerebral artery (MCA) using a 2-MHz probe (Multi-Dop T, Compumedics DWL, Singen, Germany) was performed to obtain continuous recording of MCA velocity. The probe was fixed at the temporal window with an adjustable headband and water-based ultrasound gel. Participants wore a mouthpiece and nose-clip connected to a viral filter (disposable droplet filter, MLA304, ADInstruments) and a pneumotachometer (3830 Series, Heated Linear Pneumotachometer, Hans Rudolph Inc., Kansas City, MO, USA). The pneumotachometer was connected to a three-way valve to allow for switching from room air to the rebreathing circuit (hyperoxic hypercapnia) or two-way non-rebreathing valve (isocapnic hypoxia). P_ET_O_2_ and P_ET_CO_2_ were sampled continuously via a port in the mouthpiece connected to an anaesthetic sampling line leading to a gas analyser (Respiratory Gas Analyser, ML206, ADInstruments).

### Data analysis

Graded maximal exercise test: The continuous calculation of V̇O_2_, V̇CO_2_, respiratory exchange ratio (RER), V̇_E_ and V_T_ were performed using LabChart (Version 8; ADInstruments) arithmetic and built-in Spirometry settings. All variables were exported as 15 s averages and peak V̇_E_, V̇O_2_, workload (W) and RER were obtained by finding the maximum 15 s average of each variable. HRmax (% age-predicted) was calculated using maximum HR divided by age-predicted HR max (APHRmax, APHRmax = 220-age [47]).

Hyperoxic hypercapnia (central chemoreflex assessment): Respiratory variables were extracted breath-by-breath and cardiovascular variables extracted beat-to-beat. Baseline data was averaged over the entire 5-minute period (baseline), with erroneous beats or breaths removed visually (e.g., yawn or swallow). Mean values for cardiovascular and respiratory values were obtained for the final minute of hyperventilation (hyperventilation) and final 15 s of rebreathing (peak rebreathing). Breath-by-breath V̇_E_ values from the rebreathing period were plotted against corresponding P_ET_CO_2_ values. A segmented linear regression model was used to determine the basal V̇_E_, ventilatory recruitment threshold (VRT), and central chemoreflex sensitivity using GraphPad Prism (Prism 8.0, GraphPad Software, San Diego, CA, USA). The model identifies a breakpoint whereby the slope of the line prior to the VRT = 0. The VRT is the point at which the central chemoreceptors are recruited to contribute to the drive to breath, while the slope after the VRT identifies the central chemoreceptor ventilatory sensitivity [28].

Isocapnic hypoxia (peripheral chemoreflex assessment): Respiratory variables were extracted breath-by-breath and cardiovascular variables extracted beat-to-beat. Baseline data was averaged over the entire 5-minute period, with erroneous beats or breaths removed visually (e.g., yawn or swallow). The single-breath maximal V̇_E_ value was determined and a 15 s average around this (i.e., 7.5 s prior and 7.5 s after) point calculated for respiratory variables, and a 15 s average around the same instantaneous timepoint determined for cardiovascular variables (peak). The mean value of cardiorespiratory signals was calculated for the final 1-minute of isocapnic hypoxia (end point). Peripheral chemoreflex sensitivity was calculated as the absolute increase in V̇_E_ from baseline to peak V̇_E_ expressed relative to the fall in SaO_2_.

Cerebrovascular function: Cerebrovascular reactivity to progressive hypercapnia was calculated using the method reported by Thomas et al. [38]:

$$\mathrm{Cerebrovascular reactivity}=\frac{{\mathrm{MCAv}}_{\mathrm{peak rebreathing}}-{\mathrm{MCAv}}_{\mathrm{baseline}}}{{P_{\mathrm{ET}}{\mathrm{CO}}_{2}}_{\mathrm{peak rebreathing}}-{P_{\mathrm{ET}}{\mathrm{CO}}_{2}}_{\mathrm{baseline}}}$$

Pulsatility index was calculated according to the method of Gosling and King [48] as:

$$\mathrm{Pulsatility index}=\frac{{\mathrm{MCAv}}_{\mathrm{systolic}}-{\mathrm{MCAv}}_{\mathrm{diastolic}}}{{\mathrm{MCAv}}_{\mathrm{mean}}}$$

### Statistical analysis

Normality was assessed using the Shapiro-Wilk test and visual observation. Participant characteristics were assessed using one-way analysis of variance (ANOVA) (i.e., untrained vs. endurance vs. resistance). The main effects of group (untrained vs. endurance vs. resistance) and time (phase of breathing, i.e., baseline, peak and final 15 s for hypoxia and baseline, hyperventilation and peak rebreathing for hypercapnia) and their interaction were assessed using mixed model ANOVA with repeated measures. Post-hoc analyses were performed using a t test with Bonferroni correction. Values are presented as means ± standard deviation (SD). Statistical analyses were performed using SPSS (Version 29.0, IBM Corp., Armonk, NY, USA), with significance at P < 0.05.

## Results

### Participant characteristics

Participant groups were matched for age, while resistance trained athletes were taller (P = 0.012) and heavier (P = 0.023) than untrained individuals (Table 1). Resistance trained athletes had greater handgrip strength than untrained individuals (50.9 ± 12.1 vs. 33.7 ± 10.5 kg, P = 0.003). V̇O_2_peak (mL.kg^−1^.min^−1^) was highest in the endurance trained group, followed by resistance trained, and lowest in untrained individuals (P < 0.001). Absolute V̇O_2_peak (L.min^−1^) was higher in endurance trained (P = 0.003) and resistance trained (P = 0.006) individuals compared to untrained individuals, but similar between endurance and resistance trained (P > 0.05) groups (Table 1).

**Table 1:** Participant characteristics and maximal exercise testing values.

	Untrained	Endurance trained	Resistance trained	P-value
n	10	12	12	
Age (yr)	27 ± 7	25 ± 7	29 ± 4	0.309
Female, n (%)	5 (50)	5 (42)	5 (42)	
Height (cm)	166 ± 9	175 ± 8	178 ± 10 *	**0.012**
Weight (kg)	64.8 ± 16.3	67.3 ± 6.8	80.5 ± 16.8 *	**0.023**
BMI (kg.m^−2^)	23.4 ± 3.9	22.0 ± 1.3	25.3 ± 3.4†	**0.047**
Handgrip (kg)	33.7 ± 10.5	43.2 ± 10.3	50.9 ± 12.1 *	**0.004**
V̇O_2_peak (L·min^−1^)	3.0 ± 1.3	4.5 ± 0.6 *	4.4 ± 0.9 *	**0.001**
V̇O_2_peak (mL·kg^−1^·min^−1^)	45.3 ± 9.5	66.6 ± 4.9 *	54.3 ± 5.4 * †	**< 0.001**
Peak workload (W)	122 ± 58	217 ± 59 *	218 ± 63 *	**< 0.001**
RER	1.15 ± 0.11	1.09 ± 0. 11	1.18 ± 0.05	0.070
Peak V̇_E_ (L·min^−1^)	101.2 ± 36.7	139.5 ± 18.9 *	143.5 ± 28.5 *	**0.003**
RPE	8.6 ± 1.8	9.4 ± 0.6	9.3 ± 1.0	0.312
HR max (BPM)	192 ± 13	183 ± 12	185 ± 11	0.220
HR max (%predicted)	100 ± 7	95 ± 6	97 ± 5	0.154
Resting HR (BPM)	68 ± 7	56 ± 8 *	60 ± 13	**0.024**
Resting SBP (mmHg)	113 ± 14	115 ± 8	127 ± 19	**0.045**
Resting DBP (mmHg)	69 ± 8	63 ± 5	65 ± 10	0.218
Resting MAP (mmHg)	83 ± 10	80 ± 6	86 ± 12	0.334

Values are expressed as mean±SD for continuous variables and frequency (%) for discrete variables. BMI: body mass index. RER: respiratory exchange ratio, V̇_E_: ventilation, RPE: rating of perceived exertion, HR: heart rate, SBP: systolic blood pressure, DBP: diastolic blood pressure, MAP: mean arterial pressure.* denotes P < 0.05 vs. untrained. † denotes P < 0.05 vs. endurance trained. The main effect of exercise training history (group) was examined using one-way analysis of variance (ANOVA). Where a significant effect of group was observed, post-hoc analysis was performed using t tests with Bonferroni correction.

### Cardiorespiratory responses to hyperoxic hypercapnia (central chemoreflex assessment)

**Fig. 1:**
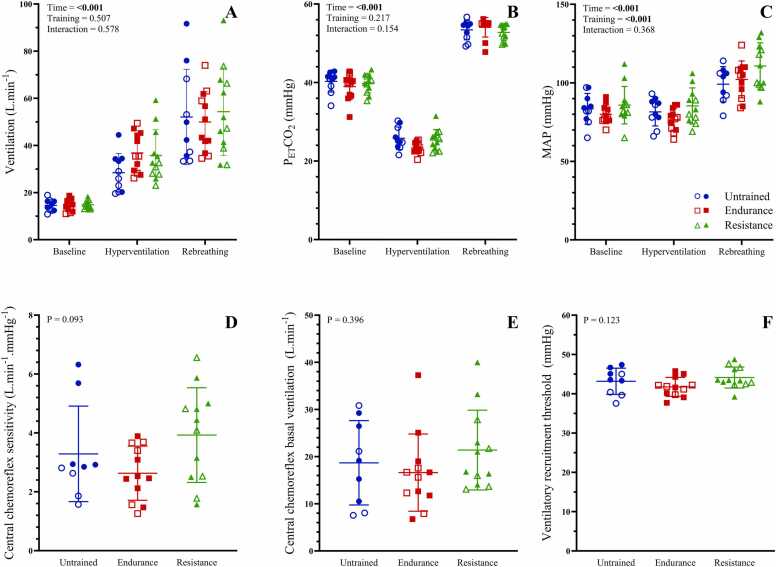
Ventilatory and mean arterial pressure responses to central chemoreflex activation. P_ET_CO_2_: end-tidal partial pressure of carbon dioxide, MAP: mean arterial pressure. Panels A-C show minute ventilation, P_ET_CO_2_ and MAP, respectively, between exercise training groups in response to hyperoxic hypercapnic rebreathing. Panels D-F show ventilatory central chemoreflex sensitivity, central chemoreflex basal ventilation, and central chemoreflex ventilatory recruitment threshold, respectively. Data are presented as means ± standard deviation (SD). Closed symbols indicate male participants, and open symbols indicate female participants.

Cardiorespiratory responses to hyperoxic hypercapnic rebreathing are shown in Fig. 1 and Table 2. By design, P_ET_CO_2_ was lowered during hyperventilation, and then increased at peak rebreathing compared to both baseline and hyperventilation (P < 0.001, Fig. 1, Panel B). V̇_E_ was increased from baseline to hyperventilation, baseline to peak rebreathing, and from hyperventilation to peak rebreathing (P < 0.001, Fig. 1, Panel A). Neither ventilatory central chemoreflex sensitivity (P = 0.093, Fig. 1, Panel D), basal ventilation (P = 0.396. Fig. 1, Panel E), nor ventilatory recruitment threshold (P = 0.123, Fig. 1, Panel F) were different between untrained, endurance trained, and resistance trained individuals.

**Table 2:** Cardiorespiratory responses to hyperoxic hypercapnia.

	Rest	Hyperventilation	Peak Rebreathing	Time	Group	Interaction
V_T_ (L)						
Untrained	0.81 ± 0.10	1.57 ± 0.56	2.00 ± 0.62	**< 0.001**	**< 0.001**	0.134
Endurance	1.01 ± 0.40	1.76 ± 0.64	2.33 ± 0.38	^a, b, c^	^e, f^	
Resistance	1.06 ± 0.30	2.51 ± 0.80	2.59 ± 0.55			
R_f_ (BPM)						
Untrained	16 ± 2	19 ± 9	24 ± 4	**< 0.001**	**0.005**	0.097
Endurance	14 ± 4	21 ± 7	20 ± 5	^b, c^	^e^	
Resistance	14 ± 4	13 ± 3	19 ± 5			
S_a_O_2_%						
Untrained	98 ± 0	99 ± 0	100 ± 0	**< 0.001**	0.641	0.936
Endurance	98 ± 0	99 ± 0	100 ± 0	^a, b, c^		
Resistance	98 ± 0	99 ± 0	100 ± 0			
P_ET_O_2_ (mmHg)						
Untrained	101 ± 7	130 ± 4‡	518 ± 22‡§	**< 0.001**	**0.001**	**< 0.001**
Endurance	102 ± 6	133 ± 3‡	453 ± 66 * ‡§	^a, b, c^	^d, f^	
Resistance	101 ± 5	132 ± 3‡	504 ± 13†‡§			
HR (bpm)						
Untrained	68 ± 7	81 ± 10	80 ± 9	**< 0.001**	**< 0.001**	0.746
Endurance	56 ± 8	75 ± 13	66 ± 13	^a, b^	^d, e^	
Resistance	60 ± 13	74 ± 15	67 ± 10			
SBP (mmHg)						
Untrained	113 ± 14	111 ± 14	133 ± 17	**< 0.001**	**< 0.001**	0.594
Endurance	115 ± 8	111 ± 10	145 ± 14	^b, c^	^e, f^	
Resistance	127 ± 19	127 ± 19	158 ± 26			
DBP (mmHg)						
Untrained	69 ± 8	67 ± 7	82 ± 8	**< 0.001**	**0.013**	0.275
Endurance	63 ± 5	59 ± 7	81 ± 11	^b, c^	^f^	
Resistance	65 ± 10	64 ± 8	87 ± 11			

Values are expressed as mean±SD for continuous variables. V_T_: tidal volume, R_f_: breathing frequency, SaO_2_%: calculated arterial oxygen saturation, P_ET_O_2_: end tidal partial pressure of oxygen, HR: heart rate, SBP: systolic blood pressure, DBP: diastolic blood pressure. The main effects of time, group and their interaction were examined using a mixed model ANOVA with repeated measures. Where a significant interaction was observed, differences identified during post hoc analysis (t tests with Bonferroni correction) are identified as * P < 0.05 vs untrained, † P < 0.0.5 vs. endurance, ‡ P < 0.05 vs. baseline, § vs. hyperventilation. Where a significant main effect of time, but no interaction, was observed, differences obtained during post hoc analysis (t-tests with Bonferroni correction) are shown as ^a^ P < 0.05 baseline vs. hyperventilation, ^b^ P < 0.05 baseline vs. peak rebreathing, ^c^ P < 0.05 hyperventilation vs. peak rebreathing. Where a significant main effect of group, but no interaction, was observed, differences obtained during post hoc analysis (t-tests with Bonferroni correction) are shown as ^d^ P < 0.05 untrained vs. endurance, ^e^ P < 0.05 untrained vs. resistance, ^f^ P < 0.05 endurance vs. resistance.

MAP (Fig. 1, Panel C) was higher during peak rebreathing compared to baseline and hyperventilation (P < 0.001). Across phases of breathing, MAP was higher in resistance trained compared to untrained individuals (P = 0.006) and endurance trained athletes (P = 0.001). Overall, V_T_ and SBP (both P < 0.05) were higher in resistance trained athletes compared to endurance athletes and untrained participants (Table 2). HR was higher in untrained participants compared to both groups of athletes (P = 0.003 and P < 0.001 compared to resistance and endurance, respectively, Table 2), while DBP was higher in resistance athletes compared to endurance trained participants (P = 0.020). R_f_ was also higher in untrained compared to resistance participants (P = 0.006), with P_ET_O_2_ lower in endurance trained individuals compared to resistance (P = 0.012) and untrained (P = 0.002) participants due to a technical issue resulting in reduced PO_2_ within the rebreathing circuit in 5 endurance trained participants. SaO_2_ was not different between groups of endurance, resistance and untrained young healthy individuals (P > 0.05). R_f_, SBP and DBP (all P < 0.001) were higher at peak rebreathing versus baseline and hyperventilation, while V_T_, SaO_2_ and P_ET_O_2_ (all P < 0.001) were increased with each stage of the rebreathing protocol. HR was lower at baseline compared to hyperventilation (P < 0.001) and peak rebreathing (P = 0.001).

### Cerebrovascular responses to hyperoxic hypercapnia

**Fig. 2:**
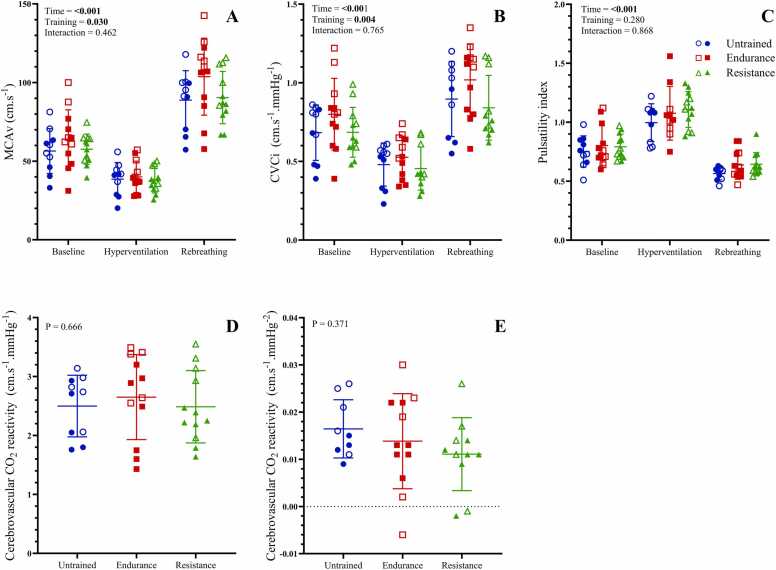
Cerebrovascular responses to hyperoxic hypercapnic rebreathing. MCAv: middle cerebral artery velocity, CVCi: cerebrovascular conductance index. Panels A-C show MCAv, CVCi and pulsatility index, respectively, between exercise training groups in response to hyperoxic hypercapnic rebreathing. Panels D and E show cerebrovascular CO_2_ reactivity calculated with MCAv and CVCi, respectively. Data are presented as means ± SD. Closed symbols indicate male participants, and open symbols indicate female participants.

**Fig. 3:**
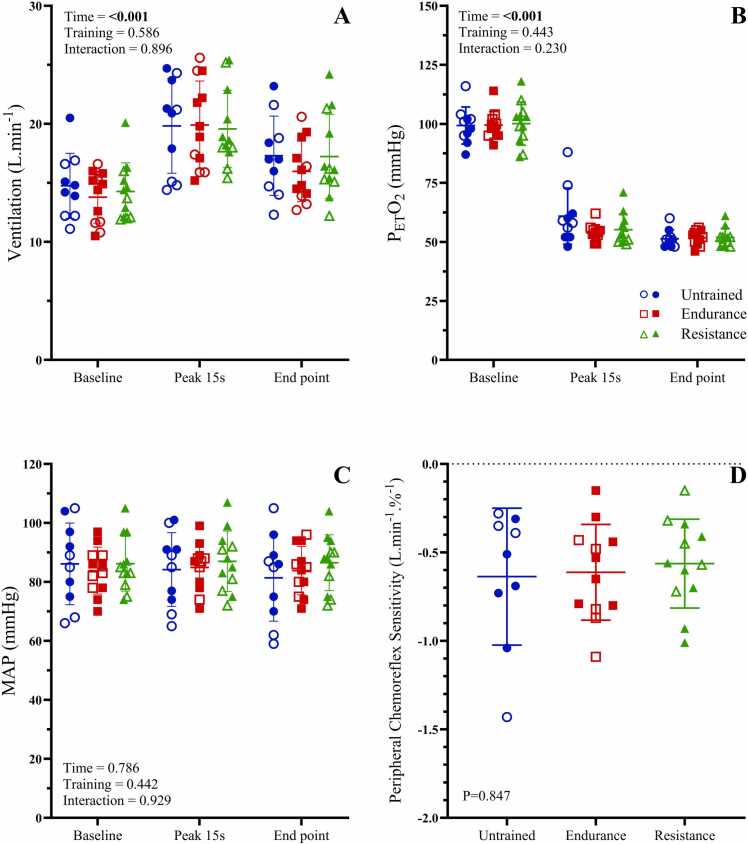
Ventilatory and mean arterial pressure responses to peripheral chemoreflex activation. P_ET_O_2_: end-tidal partial pressure of oxygen, MAP: mean arterial pressure. Panels A-C show minute ventilation, P_ET_O_2_ and MAP, respectively, between exercise training groups in response to steady-state isocapnic hypoxia and Panel D shows ventilatory peripheral chemoreflex sensitivity. Data are presented as means ± SD. Closed symbols indicate male participants, and open symbols indicate female participants.

Cerebrovascular responses to hyperoxic hypercapnia are presented in Fig. 2. As anticipated, MCAv and CVCi were lowest during hyperventilation, and increased above baseline at peak rebreathing. Additionally, endurance trained athletes had higher MCAv (P = 0.049) and CVCi (P = 0.003) than their resistance trained counterparts (P = 0.049). Pulsatility index was highest during hyperventilation, and lower than baseline at peak rebreathing. Cerebrovascular reactivity to hypercapnia was not different between groups regardless of whether it was assessed with absolute MCAv or with CVCi (P = 0.666 and P = 0.371, respectively, Fig. 3).

### Cardiorespiratory responses to isocapnic hypoxia (peripheral chemoreflex assessment)

Cardiorespiratory responses to steady state isocapnic hypoxia are shown in Fig. 3 and Table 3. Isocapnic hypoxia caused similar reductions in P_ET_O_2_ and SaO_2_, in untrained, endurance trained, and resistance trained groups. While V̇_E_ was increased from baseline during isocapnic hypoxia (P < 0.001), the magnitude of the increase was not different between groups. Ventilatory peripheral chemoreflex sensitivity was not different between untrained, endurance trained, and resistance trained groups (P = 0.847).

**Table 3:** Cardiorespiratory responses to isocapnic hypoxia.

	Rest			Peak (15 s)			End point (1 min)		Time		Group	Interaction
Tidal volume (L)												
Untrained	0.81 ± 0.13			1.00 ± 0.29			0.87 ± 0.14		**0.006**		**< 0.001**	0.855
Endurance	0.97 ± 0.43			1.24 ± 0.42			1.02 ± 0.36		^a^		^d, e^	
Resistance	1.08 ± 0.31			1.34 ± 0.39			1.27 ± 0.31					
Breathing frequency (BPM)												
Untrained	17 ± 3			19 ± 3			19 ± 3		0.166		**< 0.001**	0.947
Endurance	14 ± 4			15 ± 4			15 ± 4				^d, e^	
Resistance	13 ± 3			14 ± 4			13 ± 3					
SaO_2_%												
Untrained	98 ± 1			90 ± 4			86 ± 2		**< 0.001**		0.398	0.159
Endurance	98 ± 0			87 ± 2			86 ± 2		^a, b, c^			
Resistance	98 ± 1			88 ± 3			86 ± 2					
P_ET_CO_2_ (mmHg)												
Untrained	40 ± 3			36 ± 4			36 ± 5		**< 0.001**		0.856	0.951
Endurance	39 ± 3			36 ± 3			36 ± 3		^a, b^			
Resistance	39 ± 3			36 ± 4			36 ± 3					
Heart rate (bpm)												
Untrained	68 ± 8			78 ± 11			81 ± 9		**< 0.001**		**< 0.001**	0.844
Endurance	54 ± 8			68 ± 9			65 ± 8		^a, b^		^d, e^	
Resistance	59 ± 11			69 ± 14			69 ± 10					
Systolic blood pressure (mmHg)												
Untrained	117 ± 15			117 ± 15			113 ± 16		0.763		**0.002**	0.980
Endurance	120 ± 10			122 ± 11			120 ± 11				^e^	
Resistance	126 ± 17			128 ± 20			127 ± 18					
Diastolic blood pressure (mmHg)												
Untrained	70 ± 13			68 ± 12			66 ± 14		0.738		0.689	0.896
Endurance	66 ± 8			66 ± 7			66 ± 7					
Resistance	66 ± 6			67 ± 6			66 ± 6					

Values are expressed as mean±SD for continuous variables. V_T_: tidal volume, B_f_: breathing frequency, SaO_2_%: calculated arterial oxygen saturation, P_ET_CO_2_: end tidal partial pressure of carbon dioxide, HR: heart rate, SBP: systolic blood pressure, DBP: diastolic blood pressure. The main effects of time, group and their interaction were examined using a mixed model ANOVA with repeated measures. Where a significant main effect of time, but no interaction, was observed, differences obtained during post hoc analysis (t-tests with Bonferroni correction) are shown as ^a^ P < 0.05 baseline vs. peak, ^b^ P < 0.05 baseline vs. end point, ^c^ P < 0.05 peak vs. end point. Where a significant main effect of group, but no interaction, was observed, differences obtained during post hoc analysis (t-tests with Bonferroni correction) are shown as ^d^ P < 0.05 untrained vs. endurance, ^e^ P < 0.05 untrained vs. resistance, ^f^ P < 0.05 endurance vs. resistance

Across breathing phases, V_T_ was lower in untrained individuals compared to endurance (P = 0.026) and resistance (P < 0.001) athletes, while both R_f_ and HR were higher in the untrained group compared to both endurance and resistance participants (all P < 0.001, Table 3). SBP was higher in resistance trained athletes only compared to untrained participants (P = 0.002, Table 3). Neither P_ET_CO_2_, DBP nor MAP were different between groups of endurance, resistance and untrained young healthy individuals (all P > 0.05, Table 3). P_ET_CO_2_ was lower at peak and endpoint compared to baseline (both P < 0.001, Table 3), but HR was increased from baseline to both peak and endpoint (both P < 0.001, Table 3). V_T_ was higher at peak hypoxia compared to baseline only (P = 0.005, Table 3). All other variables (R_f_, SBP, DBP and MAP) were unchanged throughout the isocapnic hypoxic protocol (all P > 0.05, Table 3).

### Cerebrovascular responses to isocapnic hypoxia

**Fig. 4:**
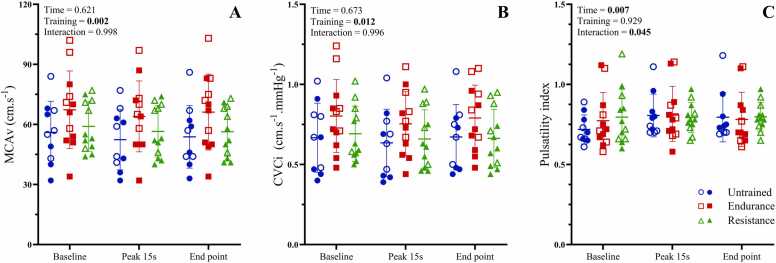
Cerebrovascular responses to steady-state isocapnic hypoxia. MCAv: middle cerebral artery velocity, CVCi: cerebrovascular conductance index. Panels A-C show MCAv, and pulsatility index, respectively, between exercise training groups in response to steady-state isocapnic hypoxia. Data are presented as means ± SD. Closed symbols indicate male participants, and open symbols indicate female participants.

Cerebrovascular responses to steady state isocapnic hypoxia are shown in Fig. 4. MCAv and CVCi were both increased in endurance trained athletes compared to resistance (P = 0.003 and P = 0.028 for MCAv and CVCi, respectively) and untrained (P = 0.24 and P = 0.037 for MCAv and CVCi, respectively) individuals. Pulsatility index was higher at peak 15 s of hypoxia compared to baseline (P = 0.007), but not different from baseline to end point or peak to end point (P > 0.05). Additionally, untrained individuals had higher pulsatility index values at peak hypoxia compared to baseline (P = 0.002), and at the endpoint compared to baseline (P = 0.008).

## Discussion

The major novel findings of the present study are: 1) chemoreflex control of breathing (central and peripheral) was not different between endurance-trained, resistance-trained and untrained young healthy individuals, and 2) endurance-trained individuals had higher MCAv and CVCi in response to hypercapnia and hypoxia, but this does not translate to increased cerebrovascular reactivity. Collectively, these findings indicate that neither endurance nor resistance training influence the chemoreflex control of breathing in young healthy adults, and that cerebrovascular function is also not affected by exercise training history.

### Exercise training and central chemoreflex sensitivity

Studies of animal models of heart failure have identified that aerobic exercise training reduces the central chemoreflex drive to breathe. This may be explained by reduced reactive oxygen species in the retrotrapezoid nucleus (RTN) [27], normalisation of the communication between the RTN and rostral ventrolateral medulla (RVLM) chemoreceptor and sympathetic neurons [49], and neuronal remodelling at central chemosensitive areas within the RTN and other cardiorespiratory areas 50, 51, 52. Based on such work, we hypothesised that central ventilatory chemoreflex sensitivity would be lower in exercise trained individuals. Contrary to our expectations, we found the ventilatory response to hyperoxic rebreathing to be similar amongst endurance-trained, resistance-trained and untrained young healthy individuals. Likewise, neither basal ventilation nor ventilatory recruitment threshold were different between the three groups. We interpret these findings as a conserved central ventilatory chemoreflex across the three cohorts studied. In agreement, previous studies have found no differences in the ventilatory response to hyperoxic hypercapnia between endurance athletes and sedentary individuals 53, 54. We extend these findings with the inclusion of a group of resistance-trained athletes. We postulated that there may be less potential for modulation of the central chemoreflex in young healthy adults with exercise training. Nevertheless, we cannot rule out the possibility that exercise training may be more effective in attenuating the central chemoreflex in clinical populations such as metabolic syndrome/obstructive sleep apnoea and heart failure, where heightened central chemoreflex sensitivity has been observed 27, 55.

### Exercise training and peripheral chemoreflex sensitivity

Beneficial reductions in peripheral chemoreflex sensitivity with exercise training in cardiovascular disease populations and animal models may arise as a result of neural plasticity within the NTS 50, 51, 52 as a site of integration for carotid body afferent signalling [56], but also due to improvements in carotid body blood flow [9]. However, while endurance training and mixed exercise interventions have been reported to elicit reductions in ventilatory peripheral chemoreflex tonicity [11] and sensitivity [14] in human studies, unchanged sensitivity 10, 12, 13 and tonicity [12] has also been documented. Therefore, the impact of aerobic or mixed exercise training on peripheral chemoreflex sensitivity is equivocal, and the effect of resistance exercise training alone is unknown. The differential effects of aerobic (increased arterial compliance, improved cerebral perfusion, and reduced arterial stiffness) 16, 17, 57) versus resistance (increased arterial stiffness 35, 58) exercise training in healthy adults may also influence common carotid artery blood flow via improved (or reduced) carotid artery reactivity [9] and thus peripheral chemoreflex sensitivity. Moreover, autonomic function measured via heart rate variability and baroreflex sensitivity is improved in young and healthy people who engage in aerobic exercise training [23], although resistance training appears to have minimal effect [24]. Considering such differential responses, we sought to determine whether exercise training modality impacts ventilatory peripheral chemoreflex sensitivity. We found the ventilatory response to isocapnic hypoxia to be similar across all three groups, suggesting that the peripheral chemoreflex is unaffected by exercise training status. A potential reason for the difference between the current study and previous work is that aforementioned human studies have assessed disease models (e.g., hypertension [11], obstructive sleep apnoea/metabolic syndrome [13], chronic obstructive pulmonary disease [12]). Additionally, a variety of methods (e.g., steady-state or progressive hypoxia, transient nitrogen inhalation) may account for the discrepancy. Nevertheless, it is feasible that the exercise training performed by participants in the present study was not a strong enough stimulus to alter peripheral chemoreflex ventilatory sensitivity despite evoking other adaptations (e.g., in heart rate and blood pressure).

### Exercise training and cerebrovascular function

Blunted cerebrovascular reactivity (i.e., CVR_CO2_) is associated with cognitive decline and risk of stroke [59]. How exercise training improves cerebrovascular function is not well understood, but may occur via increased shear stress causing increased endothelial nitric oxide synthase (eNOS) expression and nitric oxide production, acute increases in circulating catecholamines that enhance endothelial repair and angiogenesis, and reduced resting heart rate resulting in a lowered mechanical stress being placed on the cerebral endothelium [34]. While aerobic exercise training has been reported to improve CVR_CO2_ in some studies [31], no effect 60, 61 and even reduced [62] CVR_CO2_ have also been reported. Recently, Corkery and colleagues [39] showed in a cross-sectional study no difference in CVR_CO2_ between endurance trained, resistance trained or untrained adults, while Thomas et al. [38] observed with a training intervention reduced MCAv, pulsatility index and CVR_CO2_, and increased MAP, after 12-weeks of resistance exercise training but no changes following endurance training. The latter findings may indicate a protective role for resistance training to maintain global perfusion via decreased pulsatility index and increased cerebrovascular resistance [38]. However, higher pulsatility index, lower arterial compliance and increased arterial stiffness has been described in resistance trained athletes versus sedentary adults [40]. Factors influencing cerebrovascular function including central arterial stiffness and arterial compliance may be increased 35, 58 or decreased [63] depending on the intensity of resistance exercise. In contrast, aerobic exercise training has been more consistently shown to improve endothelial function and central arterial stiffness 16, 17, 37.

Herein, we extended the findings of Thomas et al. [38] and Corkery et al. [39] with the assessment of the cerebrovascular responses (including pulsatility index) to both hyperoxic hypercapnia and isocapnic hypoxia in groups of endurance, resistance and untrained young healthy individuals. It was observed that endurance trained individuals had higher MCAv during hypercapnia compared to their resistance trained counterparts, which was mirrored in their CVCi values, and driven by their reduced MAP. However, neither the CVR_CO2_ nor the pulsatility index response to hypercapnia was different in groups of endurance, resistance and untrained young healthy individuals. In response to hypoxia, aerobic trained athletes had higher MCAv and CVCi than both their resistance and untrained participants, with no difference in pulsatility index between training groups in response to hypoxia. From the present study findings, it is possible that the higher metrics of cerebrovascular function (i.e., MCAv, CVCi) observed in endurance athletes compared to their resistance and sedentary counterparts may be due to their reduced resting heart rate and therefore reduced risk of endothelial damage arising from higher mechanical stress with higher resting heart rate [34]. It is also possible that the higher MCAv and CVCi values observed in our endurance-trained cohort arise as a result of enhanced eNOS expression associated with aerobic exercise [64]. However, we did not assess carotid artery blood flow or arterial stiffness to determine whether these mechanistic pathways may have contributed to these findings. Consequently, it appears that absolute resting metrics of cerebrovascular function (i.e., MCAv and CVCi), but not cerebrovascular reactivity or pulsatility index, are elevated in those with a greater aerobic capacity (V̇O_2_). No change in cerebral/common carotid artery blood flow would be consistent with no change in peripheral chemoreflex sensitivity should peripheral chemoreflex sensitivity be attenuated by increased cerebral blood flow [36].

### Experimental considerations

The current findings should be interpreted in the light of several experimental considerations. First, the lower P_ET_O_2_ achieved during hyperoxic hypercapnia in the endurance trained group (453 ± 66 mmHg) compared to untrained and resistance trained groups (518 ± 22 mmHg and 504 ± 13 mmHg, respectively). This was the result of an initially reduced PO_2_ in the rebreathing circuit in the first five endurance trained participants. However, hyperoxia was still maintained and that P_ET_O_2_ > 150 mmHg required to dampen the peripheral chemoreceptors 46, 65. Second, five cross-fit trained individuals were included in the resistance trained group, which is an exercise mode comprising both aerobic and weight based exercise [66]. However, the mean handgrip strength values of male and female resistance trained participants achieved the inclusion criteria of > 58 kg for males and > 38 kg for females [67]. Additionally, the average V̇O_2_peak for the resistance trained group is similar to values reported in other studies of weight lifters 68, 69. Therefore, we contend that the inclusion of cross-fit trained individuals would unlikely confound our study findings. We acknowledge the relatively small sample size of the present investigation (n = 34 total, across 3 groups), and as such our findings should be interpreted considering this. Additionally, a future longitudinal study comparing endurance to resistance trained athletes would further strengthen our findings.

Finally, there has been significant recent discussion into the consideration of menstrual cycle when recruiting women for studies investigating vascular and ventilatory control 70, 71 due to the critical role of oestrogen and progesterone in the regulation of the cardiovascular system and consequential variation throughout the menstrual cycle 70, 72. The present study assessed women during the first five days of their menstrual cycle or during the placebo/non-hormone phase of oral contraceptive use as a pragmatic approach given the small number of women in each group and concerns with confounding the cardiorespiratory response to chemoreflex activation [73]. The present study is underpowered to determine sex differences within group, but equal numbers of men and women within groups and between groups were recruited.

## Conclusion

The findings of the present study indicate that central and peripheral chemoreflexes are similar between young healthy endurance- and resistance-exercise trained individuals and their untrained counterparts. Likewise, we found no differences in CVR_CO2_ and pulsatility index amongst the three groups studied. Future studies are required to determine the impact of exercise training modality on central and peripheral chemoreflex sensitivity, along with cerebrovascular function, in older adults and clinical populations, to better understand the utility of exercise prescription in these populations.

## Ethical approval

Ethical approval for the study protocol was given by the Central Health and Disability Ethics Committee, Auckland, New Zealand (20/CEN/176), and was registered with the Australian New Zealand Clinical Trials Registration (ACTRN12620001047987). All participants were provided with a comprehensive written and verbal explanation of the study protocols and provided written informed consent prior to participation in the study. The study was conducted according to the standards outlined in the latest revision of the *Declaration of Helsinki* (2013).

## Funding

This work was supported by funding from the 10.13039/501100009477Lottery Health Research/Lottery Grants Board. JFRP is funded by the Sidney Taylor Trust and is a Partridge research laureate.

## CRediT authorship contribution statement

**Thalia Babbage:** Writing – review & editing, Writing – original draft, Methodology, Investigation, Funding acquisition, Formal analysis, Data curation, Conceptualization. **Sayegh Ana L. C.:** Writing – review & editing, Methodology, Investigation. **Jui-Lin Fan:** Writing – review & editing, Methodology, Investigation. **Nicholas Gant:** Resources, Methodology. **Paton Julian F. R.:** Writing – review & editing, Supervision, Methodology, Conceptualization. **James P. Fisher:** Writing – review & editing, Supervision, Resources, Methodology, Investigation, Funding acquisition, Conceptualization.

## Declaration of Competing Interest

The authors declare the following financial interests/personal relationships which may be considered as potential competing interests: James P Fisher reports financial support was provided by Lottery Health Research. Thalia Babbage reports financial support was provided by Lottery Health Research. Julian F Paton reports financial support was provided by Sidney Taylor Trust. If there are other authors, they declare that they have no known competing financial interests or personal relationships that could have appeared to influence the work reported in this paper.

## Data Availability

Data are available from the corresponding author upon reasonable request.
